# Testicular inducing steroidogenic cells trigger sex change in groupers

**DOI:** 10.1038/s41598-021-90691-9

**Published:** 2021-05-27

**Authors:** Ryosuke Murata, Ryo Nozu, Yuji Mushirobira, Takafumi Amagai, Jun Fushimi, Yasuhisa Kobayashi, Kiyoshi Soyano, Yoshitaka Nagahama, Masaru Nakamura

**Affiliations:** 1 Institute for East China Sea Research, Organization for Marine Science and Technology, Nagasaki University, 1551-7 Taira-machi, Nagasaki, 851-2213 Japan; 2 Sesoko Station, Tropical Biosphere Research Center, University of the Ryukyus, 3422 Sesoko, Motobu, Okinawa 905-0227 Japan; 3 Research Center, Okinawa Churashima Foundation, 888 Ishikawa, Motobu, Okinawa 905-0206 Japan; 4 Present Address: Department of Biological Science and Technology, Kumamoto University, Kumamoto, 860-8555 Japan; 5 Shimane Aquarium, 1117-2 Kushiro, Hamada, Shimane 697-0004 Japan; 6 Laboratory for Aquatic Biology, Department of Fisheries, Faculty of Agriculture, Kindai University, 3327-204 Nakamachi, Nara, 631-0052 Japan; 7 National Institute for Basic Biology, Okazaki, 444-8585 Japan; 8 Noto Center for Fisheries Science and Technology, Kanazawa University, Noto-cho, 927-0552 Japan

**Keywords:** Evolution, Physiology

## Abstract

Vertebrates usually exhibit gonochorism, whereby their sex is fixed throughout their lifetime. However, approximately 500 species (~ 2%) of extant teleost fishes change sex during their lifetime. Although phylogenetic and evolutionary ecological studies have recently revealed that the extant sequential hermaphroditism in teleost fish is derived from gonochorism, the evolution of this transsexual ability remains unclear. We revealed in a previous study that the tunica of the ovaries of several protogynous hermaphrodite groupers contain functional androgen-producing cells, which were previously unknown structures in the ovaries of gonochoristic fishes. Additionally, we demonstrated that these androgen-producing cells play critical roles in initiating female-to-male sex change in several grouper species. In the present study, we widened the investigation to include 7 genera and 18 species of groupers and revealed that representatives from most major clades of extant groupers commonly contain these androgen-producing cells, termed testicular-inducing steroidogenic (TIS) cells. Our findings suggest that groupers acquired TIS cells in the tunica of the gonads for successful sex change during their evolution. Thus, TIS cells trigger the evolution of sex change in groupers.

## Introduction

Apart from fishes, vertebrates do not have a transsexual ability; however, approximately 2% of extant teleost fishes can change sex, an ability called sequential hermaphroditism^1–4^. Sex change in fishes is widely divided into three types: female-to-male (protogyny), male-to-female (protandry), and change in both directions^3–5^. Interestingly, phylogenetic and evolutionary ecological studies have recently revealed that sequential hermaphroditism in fishes is derived from gonochorism during their evolution; however, the functional mechanism of this evolution remains unclear^4–6^.

Teleost fishes exhibit a remarkable diversity among vertebrates, in not only species but also sexuality. Thus, they are widely researched as model organisms for reproductive evolutionary ecology^7^. Fishes belonging to the tribe Epinephelini in the subfamily Epinephelinae, family Serranidae, order Perciformes are commonly called groupers, and there are 163 known species belonging to 15 genera^8^. They are found in tropical to temperate coastal areas worldwide^8,9^. Groupers are one of the most described model organisms regarding reproductive evolutionary ecology and phylogeny because of their importance in fisheries and aquaculture^10,11^. In addition, groupers are a useful target species for studies on reproductive physiology due to their unique sexual characteristics. Groupers are protogynous hermaphrodites, that is, they change sex from female to male when they reach a specific size^12^. Our recent physiological investigations using a wild individual of the small honeycomb grouper (*Epinephelus merra*) as a model fish, indicated that the endogenous androgen (11-ketotestosterone; 11KT) triggers the onset of their sex change^13,14^. Cytochrome-P450-11β-hydroxylase (Cyp11b) is a steroidogenic enzyme involved in 11KT synthesis. Subsequent studies revealed that androgen-producing cells, termed testicular-inducing steroidogenic (TIS) cells, distributed in the tunica of both ovary and testis, show positive immunoreactivity against Cyp11b, and that TIS cells are the main sites of 11KT production in the ovary^15,16^.

**Figure 1:**
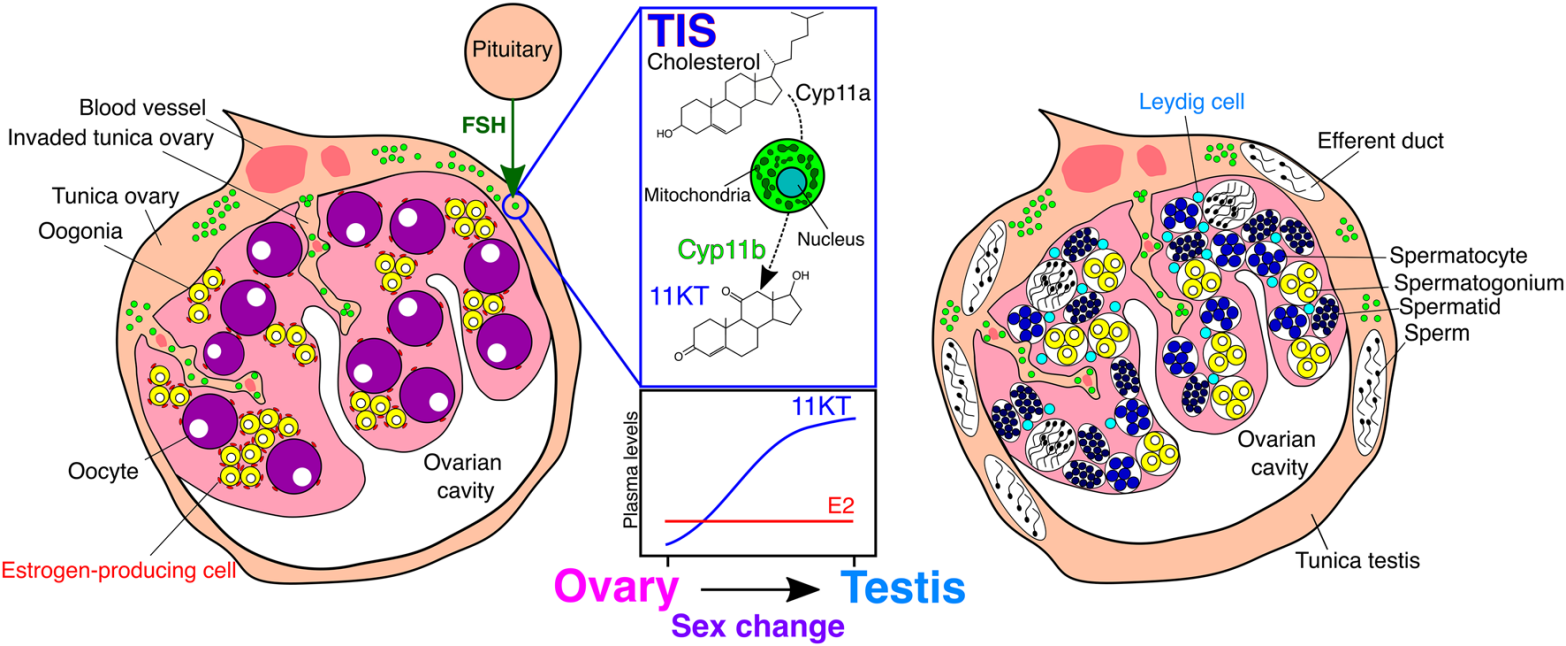
Endocrine mechanisms of sex change by TIS cells in groupers. Schematic representation of the distribution of steroid-producing cells, including TIS cells, in the gonad, and the changes in plasma sex steroid levels during the ovary-to-testis sex change in groupers, following Alam et al. (2005, 2006), Bhandari et al. (2003, 2006), and Kobayashi et al. (2010)^13–17^. *E2* estradiol-17β, *11KT* 11-ketotestosterone, *Cyp11a* cytochrome-P450-scc, *Cyp11b* cytochrome-P450-11β-hydroxylase, *FSH* follicle-stimulating hormone. The figures were constructed in Inkscape 1.0beta2 (https://inkscape.org).

In addition to these findings, we demonstrated that the pituitary follicle-stimulating hormone (FSH) most likely induces 11KT secretion in the TIS cells at the initiation of sex change^17^. Moreover, we revealed that the endocrine pathway of sex change includes TIS cells (Fig. 1). TIS cells have a typical ultrastructure of a steroid-producing cell: a large globular mitochondria-rich cell that exhibits positive immunoreactivity against cytochrome-P450-scc (Cyp11a), the key enzyme of steroidogenesis, as well as Cyp11b^15,16^. Therefore, TIS cells have the potential to independently synthesize 11KT from cholesterol, similar to the functioning of Leydig cells in the testes of gonochoristic fishes^18^. TIS cells are different from Leydig cells in not only their localization in the gonads but also their existence in the ovary. To our knowledge, functional 11KT-producing cells have not been previously reported in the ovaries of fishes. However, a recent study revealed that Malabar groupers (*Epinephelus malabaricus*) also have TIS cells in the tunica of their ovaries from the juvenile stage^19^. From these findings, we hypothesized that all groupers commonly have TIS cells in the tunica of the ovary as specific-sex change-regulating structures. In the present study, we aim to verify this hypothesis and clarify the common endocrine mechanism of sex change in groupers. We studied this by investigating the immunoreactivity of TIS cells against Cyp11b in the gonads of 7 genera and 18 species of groupers by immunohistochemistry (IHC) using the antibody against the Japanese eel (*Anguilla japonica*), Cyp11b (anti-eel-Cyp11b). In our recent studies, we already confirmed the specificity and availability of the anti-eel-Cyp11b for two species of groupers by western blotting (WB), suggesting the universal specificity of the antibody against groupers^15,19^. In addition to the groupers, we investigated the existence of TIS cells in the gonads of three species belonging to Serranidae as the nearest outgroup of groupers, which are mainly protogynous hermaphrodites. Furthermore, we investigated a species belonging to Sebastidae showing gonochorism, by IHC using commercially available antibody against medaka (*Oryzias latipes*), Cyp11b (anti-med-Cyp11b, rabbit, ab71561; Abcam)^20–22^. We have confirmed not only the specificity of the anti-med-Cyp11b for the outgroup fishes by WB but also the functional homology of the two kinds of antibodies (anti-eel and medaka-Cyp11b) by IHC. Thus, we have successfully demonstrated not only the common endocrine mechanism but also the functional evolution process of sex change triggered by TIS cells in groupers.

## Results

### Sample collection and gonadal histology

The collected species names, sample numbers, gonadal statuses, and distribution of androgen-producing cells (Cyp11b-immunoreactive cells) in the gonads, including TIS cells, are shown in Supplementary Tables S1 and S2. All gonads were examined histologically and divided into four phases: ovary, early transition, late transition, and testis (Supplementary Tables S1 and S2, and Fig S1). Histological observations revealed that an ovarian cavity was present in all testes and transitional gonads, as well as all ovaries, indicating that all testes of groupers were derived from the ovaries (Supplementary Fig S1).

### Immunohistochemistry using anti-eel-Cyp11b in grouper gonads

**Figure 2:**
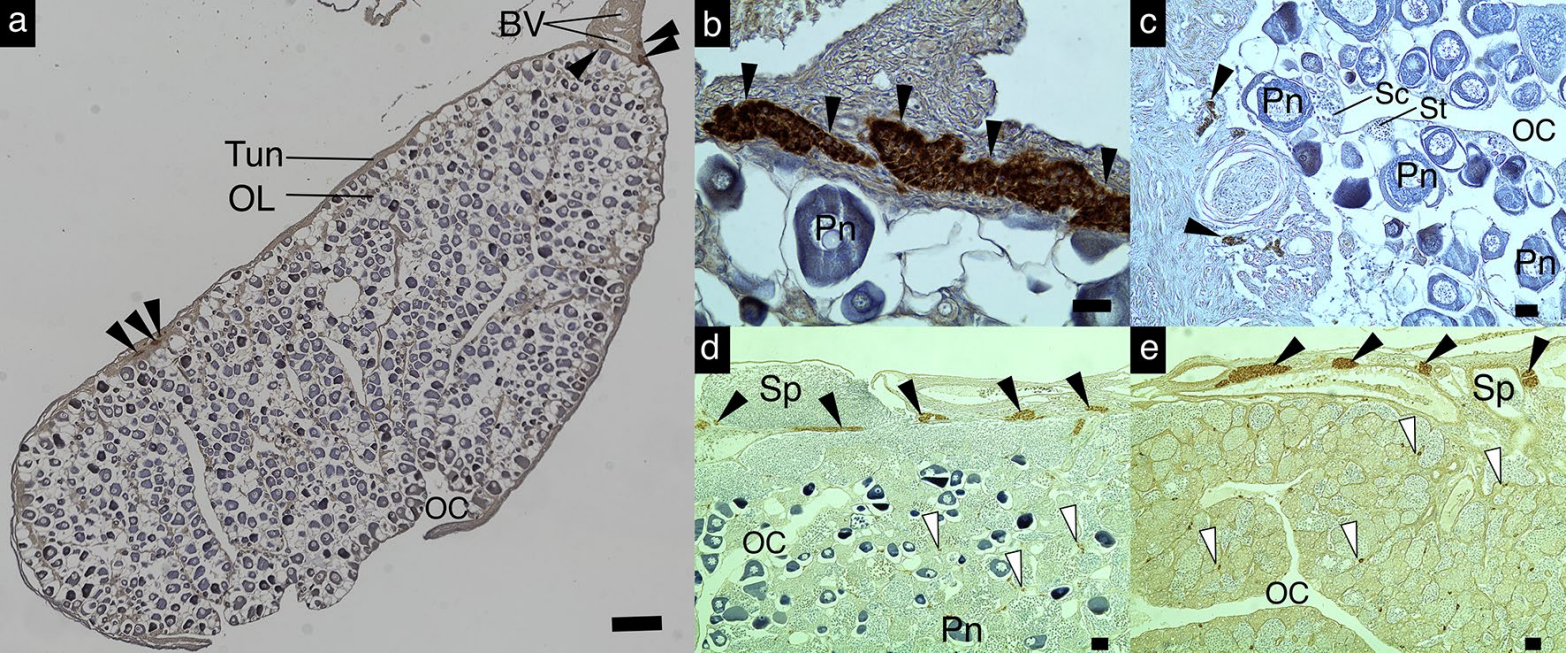
TIS cells showing immunoreactivity against Cyp11b in the tunica of the gonad of typical groupers. Immunoreactive cells against Cyp11b in the tunica of the ovary of *Epinephelus akaara* (**a**) low magnification and (**b**) high magnification, the early transitional gonad of *E. awoara* (**c**), the late transitional gonad of *E. akaara* (**d**), and the testis of *E. fasciatus* (**e**). ﻿Black and white arrow heads indicate TIS and Leydig cells, respectively. *BV* blood vessel, *OC* ovarian cavity, *OL* ovigerous lamellae, *Tun* tunica of the gonad, *Pn* perinucleolus stage oocyte, *Sc* spermatocyte, *St* spermatid, *Sp* sperm. Scale bars = 20 µm. The figures were constructed in Inkscape 1.0beta2 (https://inkscape.org).

The IHC analysis revealed anti-eel-Cyp11b-immunoreactive TIS cells in the tunica of the gonads of all grouper individuals investigated in this study, regardless of the gonadal status (Fig. 2, and Supplementary Fig S2). TIS cells were also observed in the invaded tunica tissues into the ovigerous lamellae of only three genera and six species (*Epinephelus chlorostigma*, *E. areolatus*, *E. maculatus*, *E. akaara*, *Cromileptes altivelis*, and *Plectropomus leopardus*) (Supplementary Fig S3a–d). Additionally, Leydig cells showing Cyp11b immunoreactivity were distributed in the interstitial tissues apart from the TIS cells and observed in the ovary or transitional gonads, as well as in the testes of specimens of two genera and six species (*E. chlorostigma*, *E. areolatus*, *E. maculatus*, *E. akaara*, *E. fasciatus*, and *Hyporthodus septemfasciatus*) (Supplementary Fig S3e–g). In contrast, Leydig cells were not observed in the testes or the late transitional gonads of *Cephalopholis urodeta*, *P. leopardus*, and *Variola albimarginata* (Supplementary Fig S3h).

### Specificity and functional homology between anti-eel-Cyp11b and anti-med-Cyp11b

The IHC analysis using the adjacent gonadal sections of four representative grouper species immunostained with anti-eel-Cyp11b or anti-med-Cyp11b showed the same immunoreactivity and immunolocalization (Supplementary Fig S4). The WB analysis of the gonads of the four outgroup fishes revealed that anti-med-Cyp11b recognized proteins of approximately 30 (*Sebastes ventricosus*), 37 (*Pseudanthias pleurotaenia* and *Rabaulichthys suzukii*), and 48 (*Diploprion bifasciatum*) kDa (Supplementary Fig S5).

### Immunohistochemistry with anti-med-Cyp11b in the outgroup gonads

**Figure 3:**
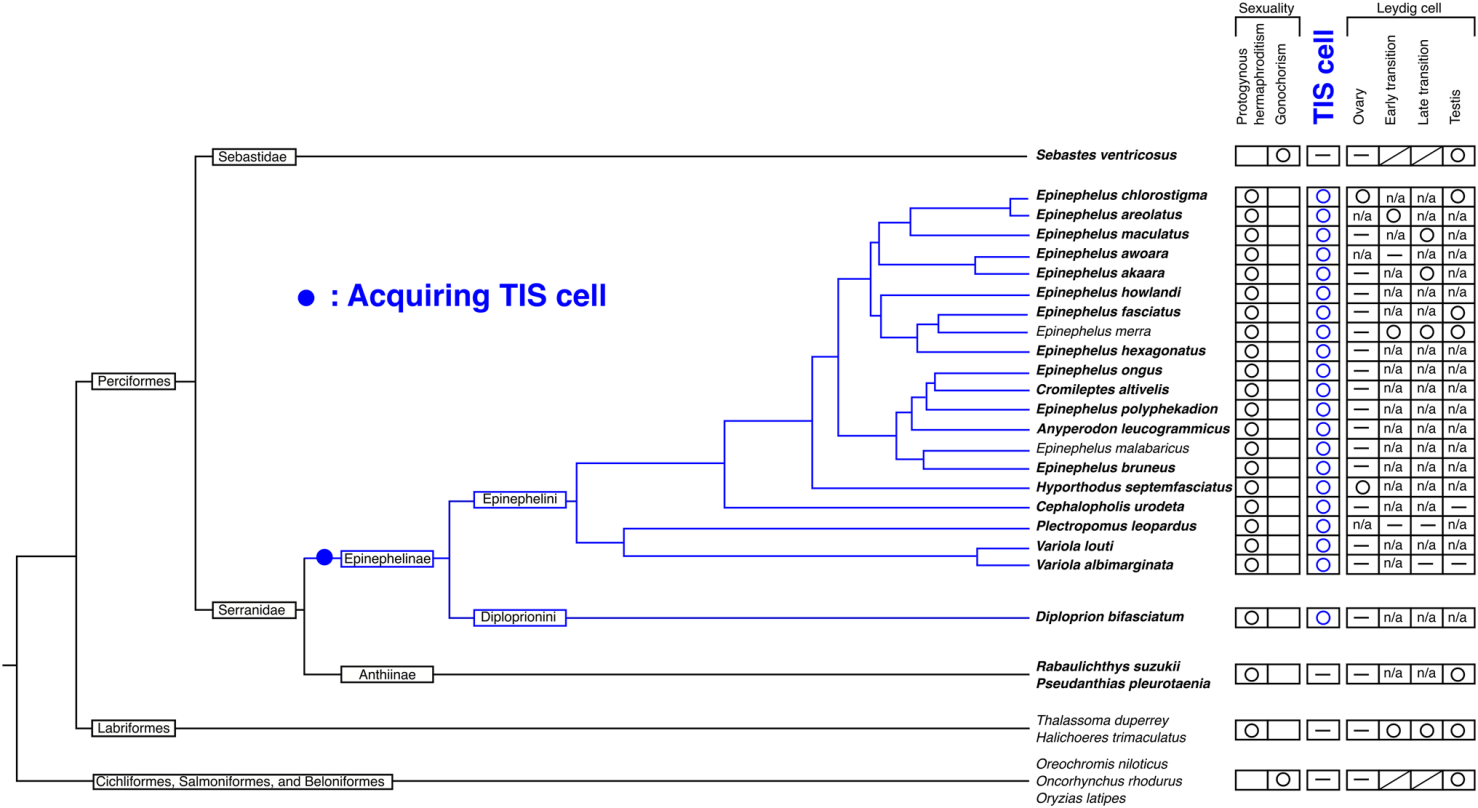
Phylogenetic position, sexuality, and existence of TIS and Leydig cells in fish gonads. The phylogenetic tree shows the evolutionary relationship among groupers, wrasses, and several gonochoristic fishes, following Ma et al. (2016) and Betancur et al. (2017)^22,24^. Species names in bold typeface indicate the results of the present study. The figures were constructed in Inkscape 1.0beta2 (https://inkscape.org).

The IHC analysis of the gonads using the anti-med-Cyp11b in the outgroups (*S. ventricosus* belonging to Sebastinae, and *P. pleurotaenia* and *R. suzukii* belonging to Anthiinae) of Perciformes revealed that there were no TIS cells in the tunica of the gonads (Supplementary Fig. S6a-f, Table S2). However, *D. bifasciatum* belonging to tribe Diploprionini, subfamily Epinephelinae, which is the nearest group of groupers, showed TIS cells in the tunica of the ovary (Supplementary Fig. S6g). The existence of TIS cells and Leydig cells, and the evolutional relationships of fishes are summarized in Fig. 3.

## Discussion

In the present study, to investigate the existence of TIS cells in the gonads of groupers, we used anti-eel-Cyp11b, which has already been proved as a universally available antibody for groupers^15,19^. Additionally, to clarify the existence of TIS cells in the gonads of the outgroup fishes, which were not covered by anti-eel-Cyp11b, we used anti-med-Cyp11b as the primary antibody for IHC. Our IHC results indicated that these two antibodies showed the same immunoreactivity in the gonads of groupers, suggesting the functional homology between these two antibodies. Although the WB analysis showed variations in the molecular weights of proteins between species due to the amino acid composition differences or phosphorylation post-translation^23^, we successfully demonstrated the specificity of anti-med-Cyp11b for the outgroup species. Thus, in the present study, we successfully investigated the existence of TIS cells (Cyp11b-immunoreactive cells distributed in the tunica of both the ovary and testis) in a wide range of Perciformes members by IHC using the two antibodies.

In this study, the sample number was limited due to the biological characteristics of groupers such as wide distribution and limited populations^9^. However, according to the latest phylogenetic constructions, the grouper species investigated in the present study are representatives from nearly all major clades of extant groupers^24^. We provide evidence that the tunica of the ovaries of all groupers investigated in this study commonly had TIS cells. These findings strongly suggest that TIS cells are common structures in the ovary of protogynous hermaphrodite groupers. Similar to the findings of a study on the honeycomb grouper, androgen has been experimentally shown to be a common and key factor for the onset of sex change in various groupers^25^. It has also been reported in *E. coioides* that endogenous 11KT triggers their natural sex change from female to male^26^. Additionally, a novel experimental study on *E. fuscoguttatus* suggested that pituitary FSH is involved in the initiation of sex change as an upstream factor, which is consistent with our previous findings regarding the honeycomb grouper^17,27^. Considering these findings, we suggest that sex change in groupers is regulated by a common endocrine mechanism: 11KT, which is secreted from TIS cells in response to pituitary FSH, triggers the onset of sex change, as previously reported in the honeycomb grouper (Fig. 1).

It is widely accepted that teleost fishes show the highest sexual plasticity among all vertebrates^28,29^. Indeed, even the phenotypic sex of gonochoristic fishes can be reversed (opposite to the genetic sex) by artificial hormone treatment during the gonadal sex differentiation period^30^. Additionally, novel experimental studies have demonstrated that the downregulation of endogenous estrogen by aromatase inhibitor treatment induces sex reversal from a genetic female to a phenotypic male; for example, in adults of the gonochoristic Nile tilapia (*Oreochromis niloticus*), Japanese medaka (*Oryzias latipes*), and zebrafish (*Danio rerio*)^31,32^. These results suggest that the gonads also maintain the steroid-dependent sexual bipotentiality during the adult stage, even in gonochoristic fishes that never undergo autonomous sex changes. The gonads of protogynous hermaphrodite groupers that are evolutionally derived from gonochoristic ancestors have been experimentally shown to have a steroid-dependent sexual bipotentiality. Both androgen addition and estrogen downregulation have been shown to successfully induce sex reversal from the ovary to testis^25,33–38^. However, the natural sex change mechanism and the evolutionary background of acquiring the autonomous transsexual ability remain unclear. In our previous studies and the present study, we demonstrated that groupers (Epinephelinae) specifically and commonly have TIS cells and that these cells regulate sex change in groupers. Additionally, we found that there are no TIS cells in the gonads of gonochoristic fishes belonging to Sebastidae, which includes groupers^21,22^. Furthermore, TIS cells are previously unknown structures in the ovaries of well-studied gonochoristic fishes^18^. These facts indicate that groupers may have acquired their autonomous transsexual ability via the TIS cells during their evolution. In conclusion, the evolution of TIS cells in groupers facilitated the successful completion of the sex change process during their evolution from gonochorism to protogynous hermaphroditism.

Conversely, we demonstrated the lack of TIS cells in the gonads of protogynous hermaphrodite fishes belonging to Anthiinae, which is the next subfamily of groupers (Epinephelinae), suggesting the possibility of a different endocrine mechanism and evolutionary pathway of sex change between Epinephelinae and Anthiinae. Moreover, it has been reported in the protogynous hermaphrodite wrasse, which lacks TIS cells, that not 11KT increase but E2 decline likely triggers their sex change^3,39^. Further studies are required to completely understand the evolutionary pathway of sex change in fishes from the viewpoint of endocrinology.

In the present study, we show that Leydig cells appear in the gonadal inner tissues, separate from the TIS cells, and that it is associated with the process of sex change in several grouper species. These findings are consistent with those of previous studies on the honeycomb grouper and saddleback wrasse (*Thalassoma duperrey*), whereby Leydig cells newly differentiate during the sexual transition^16,40,41^. We previously revealed that Leydig cells are originally derived from ovarian follicle cells after the onset of sex change^16^. On the contrary, TIS cells differentiate near the blood vessel in the tunica of the ovary by the end of ovarian differentiation during early ontogenesis, indicating that TIS cells differentiate at an earlier stage than Leydig cells during their life cycles^19^. Interestingly, the present study demonstrated that the existence of Leydig cells in the gonads differs from other grouper species. Indeed, groupers have been shown to have a supply source of androgen in the tunica of the gonads (i.e., from the TIS cells) as an alternative to Leydig cells. Based on the findings of the phylogenetic study and the present study, we hypothesize that Leydig cells might have degenerated in several ancestral groupers during evolution. However, in this study, the sample size of male groupers was too small to prove this hypothesis, and thus further study is needed. In contrast, TIS cells have universally existed in the gonads of groupers throughout their evolution, suggesting that TIS cells are among the most important cell types for the survival and evolution of groupers. Additionally, we could predict the possibility that TIS cells might be a precursor of Leydig cells; however, further research is needed to prove this hypothesis.

Furthermore, differences exist in the appearance timing of Leydig cells during the sex change process, whereby Leydig cells are visible in several species even in the ovarian stage. This suggests that Leydig cells might appear before the onset of spermatogenesis in groupers as the first characteristic of sex change. Additionally, we observed TIS cells in the invaded tunica of the gonadal medulla of certain grouper species. Despite the lack of association between the existence of invaded TIS cells and the phylogenetic relationships of groupers, these findings indicate the continued evolution of steroid-producing cells in the gonads of groupers. Interestingly, socially controlled sex change, which is well known in wrasses, has recently been demonstrated in several groupers, suggesting that groupers also have evolved the potential to change their sex in response to social cues in addition to body growth^42,43^. Further investigations are required to clarify the involvement of steroid-producing cells, including TIS cells, in the socially controlled sex change in groupers, alongside to clarify and understand the mechanism and evolution of sex change in groupers.

In conclusion, we showed that TIS cells are the functional and evolutionary trigger of sex change in groupers. Thus, groupers offer valuable physiological insights into the evolutionary process of protogynous hermaphroditism. Further research is required to clarify the mechanism of TIS cell differentiation and their functional role, other than their involvement in sex change, in groupers.

## Methods

### Ethics

All experimental procedures involving animals were conducted in compliance with the Guide for the Care and Use of Laboratory Animals (Animal-jikken-kisoku 19.6.26) of the University of the Ryukyus, the Guidelines for Animal Experimentation of the Faculty of Fisheries (fish, amphibians, and invertebrates), the Regulations of the Animal Care and Use Committee, Nagasaki University, and the ARRIVE guidelines. All experimental protocols were approved by the Animal Care and Use Committee of the Faculty of Fisheries, and the Animal Care and Use Committee of the Institute for East China Sea Research, Nagasaki University (permission # NF-0042; 15–06).

### Animals and sampling

Wild groupers were collected in Okinawa or Nagasaki Prefecture, Japan, by line fishing or were bought from anglers or fish markets in 2010–2014 and 2018–2020. The collected fishes were classified following a recent report^8^. All fishes were anesthetized with 0.05% 2-phenoxyethanol (Wako Chemicals, Osaka, Japan) before sample collection. After measuring their total body length and body weight, the specimens were euthanized by decapitation. The gonads were fixed in Bouin’s solution, embedded in paraffin, cross-sectioned to 5-µm thickness, and ﻿stained with a solution of Delafield's hematoxylin and 1% eosin, using standard methods for light microscopy. The gonadal statuses were classified according to four phases: ovary, early transition, late transition, and testis, based on the observed histological changes following the definitions provided by Bhandari et al.^13^.

### Immunohistochemical analysis

The IHC method used to analyze the expression of Cyp11b in the gonads was the same as that described by Murata et al.^19^. Briefly, 5-μm gonad sections were deparaffinized with xyline, rehydrated in graded alcohols, and finally washed with phosphate-buffered saline (PBS). The sections were then treated with 3% H_2_O_2_ for 15 min to inactivate endogenous peroxidase activity, incubated with 10% normal goat serum for 15 min to eliminate the non-specific binding, and incubated overnight with the primary antibody (diluted 1:1000–2000 in 1% BSA/PBS) in a moist chamber at 4 °C. The detection and visualization of the primary antibody were performed using the Histofine anti-rabbit immunohistochemistry kit from Nichirei (Tokyo, Japan) and diaminobenzidine per the manufacturer’s protocol.

### Western blotting

Tris-buffered saline (TBS) including protease inhibitor mix (Sigma-Aldrich, Protease Inhibitor Cocktail, Animal Component Free for use with mammalian cell and tissue extract, DMSO solution) and 0.1% Triton X-100 was added to the samples (50 mg tissue per 1 ml buffer). The samples were homogenized using a pestle on ice. After centrifugation for 20 min at 14,000 rpm and 4 °C, the supernatants were divided into aliquots and stored at − 80 °C until use. Sample aliquots were separated by SDS–PAGE under reducing conditions and blotted onto a PVDF membrane according to standard protocols^44^. Unspecific binding sites were blocked with 5% instant non-fat dry milk solution in TBST (TBS with 0.1% Tween 20). Blots were incubated with rabbit polyclonal Cyp11b1 antiserum (see above, dilution 1:1000) overnight at 4 °C. After washing with TBST three times, specific binding was detected using horseradish-peroxidase-conjugated anti-rabbit IgG (1:2000). The primary and secondary antibodies were diluted with Can Get Signal Immunoreaction Enhancer Solution (TOYOBO). Visualization was performed using EzWestBlue (ATTO).

## Supplementary Information

Supplementary Information.

## Data Availability

All data generated or analyzed during this study are included in this published article (and its supplementary information files).
