# Supplementary material for: Testicular inducing steroidogenic cells trigger sex change in groupers
Source: Sci Rep. 2021 May 27;11:11117. doi: 10.1038/s41598-021-90691-9 (PMC8160332; doi:10.1038/s41598-021-90691-9)
Supplement: Supplementary file 1 — Supplementary Information. [file 41598_2021_90691_MOESM1_ESM.pdf]

Title: Testicular inducing steroidogenic cells trigger sex change in groupers

Authors and affiliations: Ryosuke Murata, Ryo Nozu, Yuji Mushirobira, Takafumi Amagai, Jun Fushimi, Yasuhisa Kobayashi, Kiyoshi Soyano, Yoshitaka Nagahama, and Masaru Nakamura

Supplementary Information

Species name	*n*	Gonadal status				TIS cells		Leydig cells			
		Ov	ET	LT	Tes	Tun	In. Tun	Ov	ET	LT	Tes
Epinephelus. chlorostigma	**2**	**1**	**-**	**-**	**1**	**2**	**2**	**1**	**n/a**	**n/a**	**1**
E. areolatus	**3**	**-**	**3**	**-**	**-**	**3**	**1**	**n/a**	**2**	**n/a**	**n/a**
E. maculatus	**4**	**2**	**-**	**2**	**-**	**4**	**3**	**-**	**n/a**	**2**	**n/a**
E. awoara	**3**	**-**	**3**	**-**	**-**	**3**	**-**	**n/a**	**-**	**n/a**	**n/a**
E. akaara	**6**	**3**	**-**	**3**	**-**	**6**	**3**	**-**	**n/a**	**3**	**n/a**
E. howlandi	**5**	**5**	**-**	**-**	**-**	**5**	**-**	**-**	**n/a**	**n/a**	**n/a**
E. fasciatus	**10**	**7**	**-**	**-**	**3**	**10**	**-**	**-**	**n/a**	**n/a**	**3**
E. hexagonatus	**1**	**1**	**-**	**-**	**-**	**1**	**-**	**-**	**n/a**	**n/a**	**n/a**
E. ongus	**5**	**5**	**-**	**-**	**-**	**5**	**-**	**-**	**n/a**	**n/a**	**n/a**
Cromileptes altivelis	**1**	**1**	**-**	**-**	**-**	**1**	**1**	**-**	**n/a**	**n/a**	**n/a**
E. polyphekadion	**2**	**2**	**-**	**-**	**-**	**2**	**-**	**-**	**n/a**	**n/a**	**n/a**
Anyperodon leucogrammicus	**3**	**3**	**-**	**-**	**-**	**3**	**-**	**-**	**n/a**	**n/a**	**n/a**
E. bruneus	**4**	**4**	**-**	**-**	**-**	**4**	**-**	**-**	**n/a**	**n/a**	**n/a**
Hyporthodus septemfasciatus	**3**	**3**	**-**	**-**	**-**	**3**	**-**	**1**	**n/a**	**n/a**	**n/a**
Cephalopholis urodeta	**5**	**4**	**-**	**-**	**1**	**5**	**-**	**-**	**n/a**	**n/a**	**-**
Plectropomus leopardus	**2**	**-**	**1**	**1**	**-**	**2**	**2**	**n/a**	**-**	**-**	**n/a**
Variola louti	**2**	**2**	**-**	**-**	**-**	**2**	**-**	**-**	**n/a**	**n/a**	**n/a**
V. albimarginata	**5**	**3**	**-**	**1**	**1**	**5**	**-**	**-**	**n/a**	**-**	**-**

Supplementary Table S1. Groupers species names, sampling size (*n*), gonadal statuses (Ov, ovary; ET, early transitional stage; LT, late transitional stage; Tes, testis), Cyp11b-immunoreactive cell existence in the tunica of the gonads (Tun), in the invaded tunica (In. Tun) indicating the TIS cells, and in the interstitial tissues indicating the Leydig cells. The groupers species names are arranged evolutionally (i.e., the more derived species are listed first in the table), and the double lines represent the main clades, following Ma et al., 2016 [23]. The table was constructed in Microsoft Office Excel for Mac.

Species name	*n*	Gonadal status				TIS cells		Leydig cells			
		Ov	ET	LT	Tes	Tun	In. Tun	Ov	ET	LT	Tes
Sebastes ventricosus	**5**	**3**			**2**	**-**	**-**	**-**			**2**
Pseudanthias pleurotaenia	**3**	**2**	**-**	**-**	**1**	**-**	**-**	**-**	**n/a**	**n/a**	**1**
Rabaulichthys suzukii	**5**	**1**	**1**	**-**	**3**	**-**	**-**	-	**-**	**n/a**	**3**
Diploprion bifasciatum	**1**	**1**	**-**	**-**	**-**	**1**	**-**	**-**	**n/a**	**n/a**	**n/a**

Supplementary Table S2.

Outgroup species names, sampling size (*n*), gonadal statuses (Ov, ovary; ET, early transitional stage; LT, late transitional stage; Tes, testis), Cyp11b-immunoreactive cell existence in the tunica of the gonads (Tun), in the invaded tunica (In. Tun) indicating the TIS cells, and in the interstitial tissues indicating the Leydig cells. The table was constructed in Microsoft Office Excel for Mac.

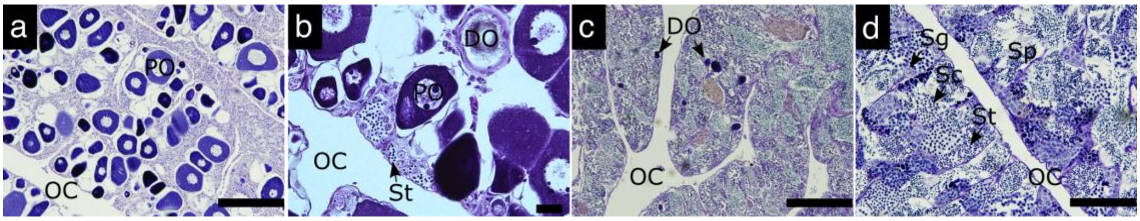

Supplementary Figure S1. Histological images of the four stages of the gonads in groupers. Ovary in *Epinephelus fasciatus* (a), early transitional stage gonad in *E. awoara* (b), late transitional stage gonad in *Variola albimarginata* (c), and testis in *Cephalopholis urodeta* (d). PO, peri-nucleolar stage oocyte; OC, ovarian cavity; Sg, spermatogonia, Sc, spermatocyte; St, spermatid; Sp, sperm; DO, degenerating oocyte. Scale bars = 20 μm in

(b), 200 µm in (a and d), and 500 µm in (c). The figures were constructed in Inkscape 1.0beta2 (https://inkscape.org).

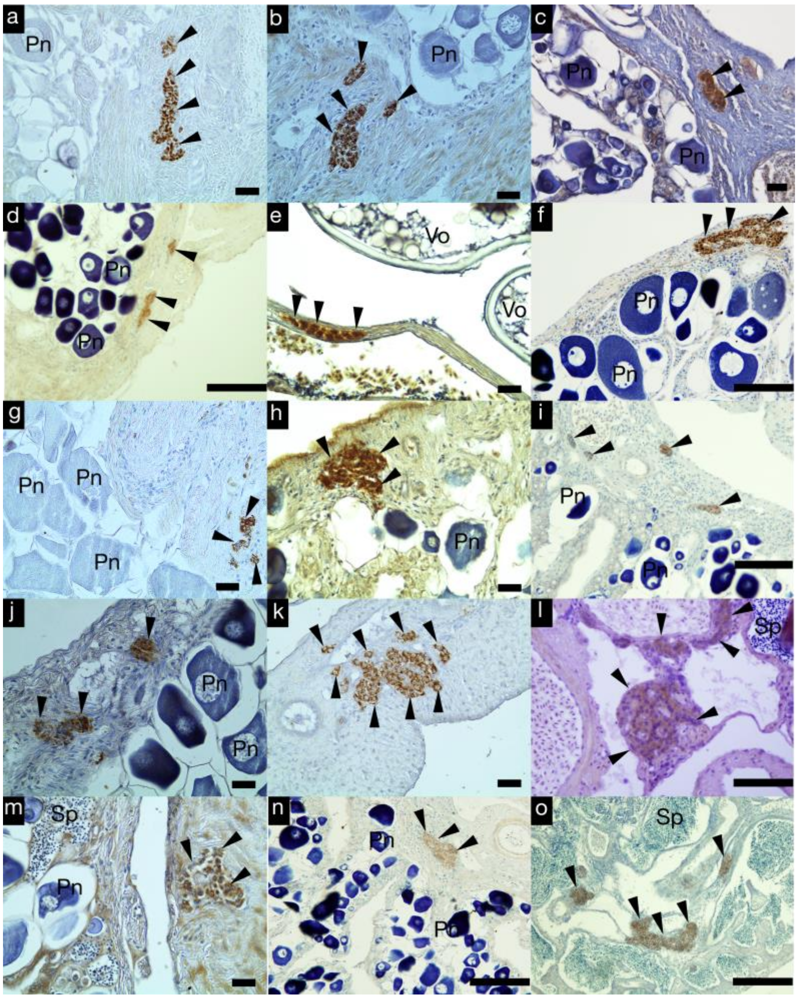

Supplementary Figure S2. TIS cell showing immunoreactivity against Cyp11b in the tunica of the gonad of *Epinephelus chlorostigma* (a), *E. areolatus* (b), *E. maculatus* (c), *E. howlandi* (d), *E. hexagonatus* (e), *E. ongus* (f), *Cromileptes altivelis* (g), *E. polyphekadion* (h), *Anyperodon leucogrammicus* (i), *E. bruneus* (j), *Hyporthodus septemfasciatus* (k), *Cephalopholis urodeta* (l), *Plectropomus leopardus* (m), *Variola louti* (n), and *V. albimarginata* (o). Arrows indicate positive signals (TIS cells). Pn, perinucleolus stage oocyte; Sp, sperm; Vo, vitellogenic oocyte. Scale bars = 20 µm in (a–

c, e, g, h, j, k, and m), 50 μm in (l), 100 μm in (d, f and i), and 200 μm in (n and o). The figures were constructed in Inkscape 1.0beta2 (https://inkscape.org).

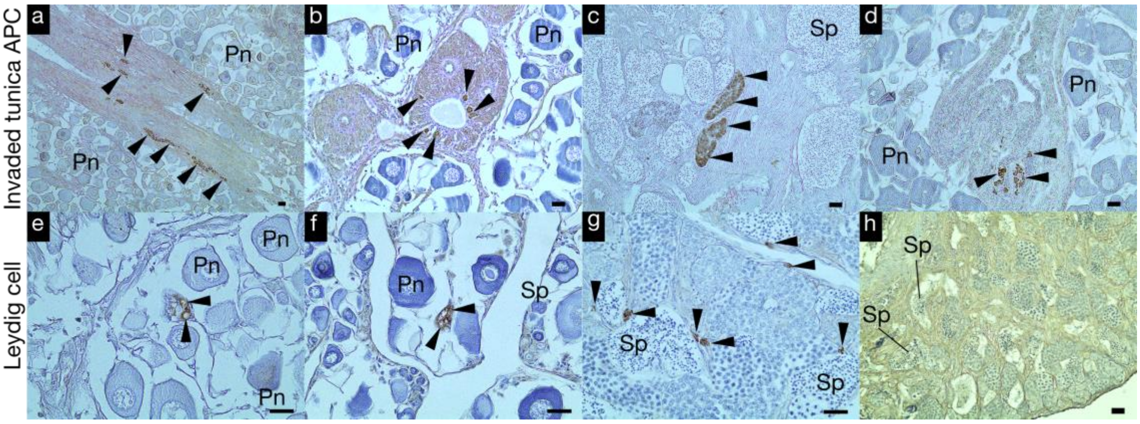

Supplementary Figure S3. Immunoreactive cells against Cyp11b in the invaded tunica of the gonads (TIS cells) of *Epinephelus chlostigma* (a), *E. areolatus* (b), *E. maculatus* (c), and *Cromileptes altivelis* (d). Immunoreactivity against Cyp11b in the interstitial gonads (Leydig cells) of *E. chlostigma* (e), *E. areolatus* (f), *E. maculatus* (g), and *Plectropomus leopardus* (h). Arrows indicate positive signals. Pn, perinucleolus stage oocyte; Sp, sperm. Scale bars = 20 μm. The figures were constructed in Inkscape 1.0beta2 (https://inkscape.org).

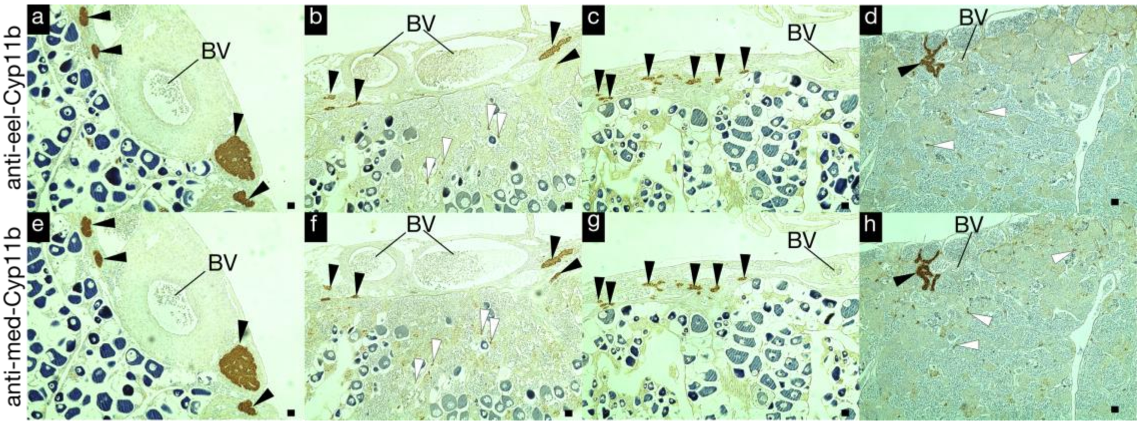
anti-eel-Cyp11b
anti-med-Cyp11b
a
b
c
d
e
f
g
h
BV

Supplementary Figure S4. Immunohistochemical analysis of the gonadal adjacent sections of 4 representative grouper species (a and e, *Epinephelus fasciatus*; b and f, *E. akaara*; c and g, *E. bruneus*; d and h, *E. merra*) using anti-eel-Cyp11b (a-d) and anti-medaka-Cyp11b (e-h). Black and white arrow heads indicate TIS and Leydig cells, respectively. BV, blood vessel. Scale bars = 20 µm. The figures were constructed in Inkscape 1.0beta2 (https://inkscape.org).

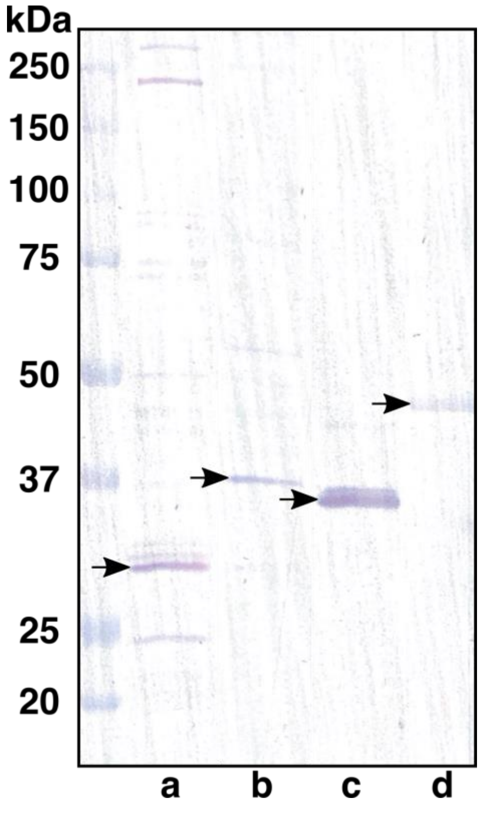

Supplementary Figure S5. Western blot analysis of *Sebastes ventricosus* (lane a), *Pseudanthias pleurotaenia* (lane b), *Rabaulichthys suzukii* (lane c), and *Diploprion bifasciatum* (lane d) gonadal proteins using anti-medaka Cyp11b antiserum. Specific signals are indicated by arrows on the left of each lane. The molecular masses of protein standards are indicated on the left. The figures were constructed in Inkscape 1.0beta2 (https://inkscape.org).

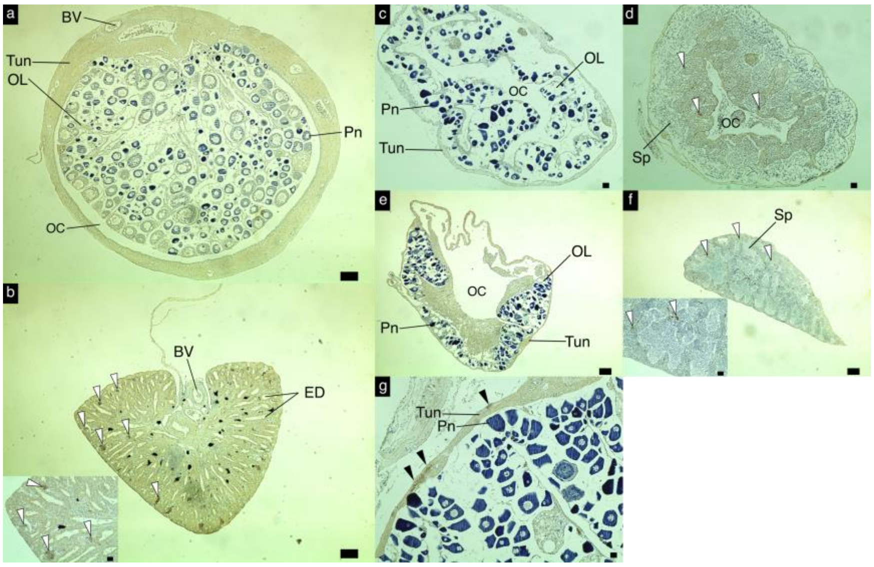

Supplementary Figure S6. Immunoreactive cells against Cyp11b in the gonads of *Sebastes ventricosus* (a, ovary; b, testis), *Pseudanthias pleurotaenia* (c, ovary; d, testis), *Rabaulichthys suzukii* (e, ovary; f, testis), and *Diploprion bifasciatum* (g, ovary). Black and white arrow heads indicate TIS and Leydig cells, respectively. BV, blood vessel; EF, efferent duct; OC, ovarian cavity; OL, ovigerous lamellae; Tun, tunica of the gonad; Pn,

perinucleolus stage oocyte; Sp, sperm. Scale bars = 20 μm. Inset figures indicate high-magnification images. The figures were constructed in Inkscape 1.0beta2 (https://inkscape.org).
